# Use of Cognitive Interviews in the Development of a Survey Assessing American Indian and Alaska Native Adult Perspectives on Genetics and Biological Specimens

**DOI:** 10.3390/ijerph21091144

**Published:** 2024-08-29

**Authors:** Vanessa Y. Hiratsuka, Julie A. Beans, Christie Byars, Joseph Yracheta, Paul G. Spicer

**Affiliations:** 1 Southcentral Foundation, Anchorage, AK 99508, USA; jbeans@southcentralfoundation.com; 2 Chickasaw Nation, Ada, OK 74820, USA; christie.byars@chickasaw.net; 3 Native BioData Consortium, Eagle Butte, SD 57625, USA; joseph@nativebio.org; 4 Center for Applied Social Research, University of Oklahoma, Norman, OK 73072, USA; paul.spicer@ou.edu

**Keywords:** genetic research perceptions, survey development, cognitive interviews, American Indian and Alaska native people

## Abstract

The cognitive interview process is a method to validate a survey instrument’s face validity and enhance confidence in item interpretation, as well as a method to engage communities in the research process. Trained American Indian and Alaska Native (AIAN) interviewers conducted retrospective cognitive interviews at three AIAN communities to assess the item quality of a 131-item survey item that measures AIAN knowledge and attitudes on genetics and biological specimens. A cognitive interview process was used to assess cultural consonance, thought processes used when considering survey instructions, items and responses, and language preference of survey items in the development of a survey to assess public knowledge and attitudes on genetics. Content analysis was used to analyze interview data. Survey instructions, items and scales generated no cognitive difficulties. The participants noted being unfamiliar with terminology used to describe genetic and biological specimens. In several cases, the participants’ written response in the survey and verbal response in the interview did not align. A resultant 52-item survey for use in AIAN communities was finalized. Cognitive interviewing is resource-intensive; however, ignoring community engagement during survey development results in inappropriate interpretations about culturally diverse populations such as AIAN peoples.

## 1. Introduction

Past research with the American Indian and Alaska Native (AIAN) communities demonstrated interest in genetic research when individual and group harm protections are present [1,2,3,4]. Additionally, genetic research must address areas of known disparities where potential health benefits are clear [2,5]. Clear communication regarding genetics and biological specimens in AIAN communities is a key component for successful outcomes [3,6].

An aspect of protecting the community is including the community voice throughout the project [1]. Tribal communities and their university partners have successfully implemented a series of pharmacogenetic efforts [3,6,7,8,9,10,11,12,13], as well as genetic analysis using AIAN cohort study specimens [14,15,16,17,18,19,20,21].

As genetic, pharmacogenetic and precision medicine research expands within Tribal communities, careful attention is needed to implement robust informed consent processes, determine data use and stewardship processes, develop plans for dissemination of findings, and allow for both human subjects review and Tribal oversight and approval of the research [22,23,24,25]. As Tribes decide to participate in a wide range of genomic research protocols, Tribal leaders and Tribal citizens have been examining (1) if it would be beneficial for the community for genomic research to take place, and (2) if so, how it should be carried out [8,23,24,25,26,27]. The Tribal Collaboration Working Group (TCWG) of the All of Us Research Program Advisory Panel guidance to senior leadership of the All of Us Research Program overarching principles for engagement with Tribal communities includes respect for Tribal sovereignty, acknowledgement of historical transgressions, importance of engagement with Urban Indian leaders, and a need for continued bi-directional engagement [28].

### 1.1. Center for the Ethics of Indigenous Genomic Research

AIAN individuals make up approximately 1.7% of the U.S. population [29]. The AIAN population is highly heterogeneous—there are a total of 347 federally recognized Indian Tribes within the contiguous 48 states and 227 federally recognized Tribal entities within the state of Alaska that comprise the 574 federally recognized Indian Tribes of the United States [30]. There are also a number of AIAN Tribes that do not have federal recognition, or their past federal recognition was terminated [30]. AIAN Tribes and AIAN individuals may have different views on biomedical research due to differing experiences with research and clinical care, and state and federal policy implementation [24].

The Center for the Ethics of Indigenous Genomic Research (CEIGR), a National Institutes of Health-funded Center for Excellence in ethical, legal, and social implications research [31,32], sought to develop and conduct a survey for use in AIAN communities nationally to explore public knowledge and attitudes on genetics and biological specimens among AIAN people. CEIGR is based on the collaborative working relationship between researchers at the University of Oklahoma and three research groups based in AIAN communities led by Indigenous researchers: the Chickasaw Nation; Missouri Breaks Industries Research Inc. (Eagle Butte, SD, USA), an AI–owned private research organization; and Southcentral Foundation, an AN Tribal health organization based in Anchorage, Alaska [31,32]. The AIAN community based and placed research teams within CEIGR collaborate with the AIAN Tribe or Tribes in their region to develop and implement health research of consequence to their populace [31,33].

### 1.2. Preliminary Item Development

CEIGR members sought to develop a survey on the attitudes and knowledge of genetics and biological specimens in three AIAN communities to inform the development of briefing materials for future public deliberation activities. Part of conducting work in AIAN communities is adhering to the requirement to include the community in all phases of the research project [34]. In consideration of the development and implementation of an AIAN community member survey, the CEIGR partners discussed empirical bioethics research best practices on survey development. An in-person gathering of an expert panel was held to discuss topics for inclusion. The expert panel represented the fields of anthropology, communication, public health, biology, genetics, Native American studies, and biomedical ethics. Expert panelists discussed potential scales and item constructs. The process resulted in the development of a preliminary survey comprising 137 items using scales that were previously tested in other populations (e.g., conduct of research; involvement in research; genetic testing risk and benefits; direct to consumer testing) [34,35] and scales designed by the CEIGR team specifically to address AIAN community concerns (e.g., personal beliefs; perceptions of researchers and research regulations; benefits and harms of research; demographics). The items were adjusted based on an initial review for readability. In addition, site-specific questions were developed, which include demographic questions. A seven-point Likert scale with response anchors (i.e., 1 = strongly disagree, 7 = strongly agree) was used for all responses to avoid respondents needing to move between multiple response types within the survey.

### 1.3. Cognitive Interviewing

The CEIGR AIAN site and university leadership decided to incorporate AIAN community member viewpoints to assess the content validity of survey items through cognitive interviews with AIAN community members. Cognitive interviewing (CI) is the administration of draft survey questions while collecting additional verbal information about the survey responses, which is used to evaluate the quality of the response or to help determine whether the question is generating the information the author intends [36,37,38]. CI is an approach that is used to evaluate sources of response error in survey instructions, survey items, and corresponding response options [39,40]. The CI approach is recommended in the early phase of survey development to gain an understanding of the cognitive processes survey respondents use while engaging with survey instructions and items [40,41,42,43].

In this study, we used a retrospective cognitive interview process [37,44]. The main purpose of the cognitive interviews was to determine community acceptability of the item and if acceptable, to improve the item quality by uncovering the cognitive processes AIAN primary care patients used to answer the question items. Emphasis for adaptation was placed on cultural relevance, comprehensibility, and language preferences. Within the CEIGR survey, we assessed the comprehensibility of survey instruction, questions, terms, and responses, and to explore the personal experiences, the given responses were based on the type of information the respondents applied while answering the items.

## 2. Materials and Methods

### 2.1. Participants, Setting and Procedures

CIs were conducted in three AIAN primary care settings: the Chickasaw Nation in Oklahoma, the Cheyenne River Reservation in South Dakota, and at the Alaska Native Medical Center in Anchorage, Alaska. Recruitment occurred in AIAN-serving primary care clinic lobbies using convenience sampling of individuals who were eligible for AIAN health services, aged 18 years or older, and who had received health care at the facility.

### 2.2. Training

A two-day training for study staff occurred on 22–23 February 2018 in Anchorage, Alaska. The AIAN study staff were trained in the theory and practice of conducting retrospective cognitive interviews, the use of verbal probing, and think-aloud processes by an AI public health researcher (VYH). The training included a review of the survey that was to be given to the participants, practice of the retrospective cognitive interview techniques, practice of note taking and data entry, and discussion on analysis. In addition, the data collection team from the three sites attended bi-weekly conference calls during data collection to provide updates on data collection, discuss processes to ensure each site completed data collection according to protocol, and share ideas on addressing issues as they arose. CI support was provided via email and phone calls throughout the data collection process.

### 2.3. Ethics Review

Institutional review board approval and community approval/Tribal approval was obtained at all sites prior to the conduct of research. Additionally, community-level review of all dissemination materials including this manuscript was carried out at all sites prior to presentation or publication.

### 2.4. Recruitment

At each of the three study locations, up to 25 individuals were recruited in person at a recruitment table in the lobby of primary care clinic locations. During recruitment, research team members explained the purpose of the cognitive interviews as well as other specifics of participation (e.g., length of survey, voluntary participation) and were available for questions. Interested individuals were provided information about the interview process, contact information for research staff if they had questions prior to the interview, and scheduled for an in-person interview.

### 2.5. Data Collection/Cognitive Interviewing Process

An informed consent form was provided to each participant prior to the interview. The participants were provided time to review the consent form; the researcher reviewed the consent form with the participant and then allowed time for questions. Each participant provided verbal consent.

The participants completed the 131-item draft survey. Then, retrospective cognitive interviews were conducted using a standardized interview guide on a subset of questions. By allowing participants to complete the full survey, we were able to assess the overall survey experience and discuss issues in self-administration. To allow for between-subject comparisons, each question received up to three reviews at each site. The retrospective administration replicated the conditions of survey administration in practice. With participant permission, the interviews were audio-recorded. Interviews took place at administrative offices associated with clinic locations on weekdays during working hours.

During the CI, verbal probing and a retrospective think-aloud process was used. A structured set of verbal probes was used for all items. The probes were as follows: (1) What do you think about this question? (2) What does this question mean to you? (3) Was it difficult to answer? If yes, what made it difficult? (4) Was it offensive or insensitive in any way? If yes, is there another way we could ask it that would be better? Interviewers took detailed notes during the interview with an emphasis on documentation of responses related to face validity. Face validity was assessed by directly asking the participant about the question and discussing their response to the question given their description of the question. Following each interview, the participants received a gift card in the amount of USD 40 as compensation for their time.

Immediately after the interview, each interviewer reviewed hand-written notes taken during the interview and checked notes with audio recordings. Notes were inputted into a Microsoft Excel spreadsheet for site-specific and cross-site analysis.

### 2.6. Analysis

Analysis indicated potential sources of response error, issues with item interpretation, and face validity using an adjusted form of Tourangeau’s model of cognitive processing [44]. This model describes the survey response process as involving comprehension, retrieval of information, judgment or estimation, and selection of a response to the question [37,40]. One researcher (VYH) conducted site-specific and cross site analysis in close collaboration with the site-based researchers (JAB, CB, JY). The data collection team from all sites reviewed issues per item via video teleconference. Review within sites and across sites for cognitive processing assessed for potential sources of response error, issues with item interpretation, and face validity using an adjusted form of Tourangeau’s model of cognitive processing [38]. A seven-step analytic process unique to this study was used.

1. Site-based researchers reviewed the notes entered in Microsoft Excel from each cognitive interview.
2. Each instruction, item, and/or scale was assessed to determine if it was working as intended.
3. Problems with each instruction, item and/or scale were reviewed to determine if they were cross-site or site-specific issues
4. Revision suggestions for each issue were determined.
5. A presentation of CI results was made to each site, reviewing site-specific issues and discussing draft revisions.
6. A presentation of CI results was made to the CEIGR expert panel, reviewing cross-site issues and responses previously discussed in step five.
7. Site leads and CEIGR leadership decided on revised items to be used in the survey that would be fielded at their local site. Items that were selected at all three sites were considered as the 52-item CEIGR cross-site survey.

## 3. Results

A total of 52 AIAN adults participated in cognitive interviews across the three partner sites from March 2018 to May 2018. The overall sample distribution was unequally distributed, with sites A and B contributing 40% of the sample each and site C contributing 20%. Overall, the sample was equally distributed between male and female participants, who considered themselves to be AIAN alone (86%) and were not Latino (90%); the participants were relatively evenly distributed by decile between the ages of 25 and 65 years, the majority of respondents had children (84%) and had grandchildren (55%), had a level of education of some college or higher (67%), and the majority lived in rural areas. Most participants (79%) reported having overall health that was good or better and had received healthcare at a Tribal health facility ranging from just two months ago to a lifetime of care at that location. Overall, participants in the sample were nearly equally distributed between having and not having previous participation in research. Most participants (92%) had not shared a biological sample in a research study nor had they shared a biological sample in a biobank as part of participation in a research study. Finally, only 15% of the overall sample had sought information about genetics; however, a third of the Alaska-based sample had sought out genetic information.

Participants understood the complex topics of genetics and biological specimens, and found the topics very interesting. All participants were engaged throughout the interview process, which took up to two and a half hours. The instruction, items, and scales generated no cognitive problems (e.g., recall, decision-making capacity). The participants requested wording changes in some scale instructions and items (Table 1). Problems noted included participants being unfamiliar with some terms used describing genetic and biological specimens. When a participant was unfamiliar with a term, their response was either randomly selected, a neutral response, or the participant did not elect to select a response. In several cases, the participants’ written response in the survey and verbal response in the interview did not align, indicating a problem with the item.

### 3.1. Participant Response to Cognitive Interviewing

The participants commented that they appreciated the opportunity to provide input and be a part of the survey development. The participants emphasized the importance of gathering community member perspectives in the development of health surveys. Project staff were thanked by the participants for taking the time to ask these important questions of them and to take into consideration their recommendations to improve the wording and comprehension of the instrument for use in Tribal communities. Several participants asked if the cognitive interviewing process was used for all clinical instruments at the Tribal health facility. A few noted that the process of member checking was indicative of self-determined Tribal health services.

### 3.2. Science and Society

The participants were asked their level of agreement for five statements about their views on science and the scientific method. Items in the science and society section were as follows: “The scientific process is the only valid and reliable way to understand nature”, “Scientific evidence can be interpreted to fit opposing points of view”, “Intuition can provide an understanding of nature as valid as that of science”, “The results of scientific research will always be significantly affected by the values held by the researcher”, and “Science and advanced technologies can solve almost all of society’s problems”. The participants had wide variability in their understanding of each of the statements in this section of the instrument. Frequently, the participants noted that concepts like “nature”, “science”, “scientific evidence”, “society’s problems” and “scientific research” could be defined in many ways and were not sure if their definition was the same as the survey intended. Some participants mentioned that their lack of understanding caused them to reply with a neutral response, as one participant stated “I mean what scientific evidence are they using. It’s a tricky question. What does that statement mean?”. Given the need to define multiple terms in the statements and the use of a neutral response when participants were uncertain of the item’s meaning, the items on science and society were removed.

### 3.3. Direct-to-Consumer Ancestry Testing

The participants were asked their level of agreement for six statements about their views on direct-to-consumer ancestry testing. Prior to the items, a statement was provided: “Direct-to-consumer ancestry companies that provide the public, for a fee, with genetic screening results that contain information about certain health markers that may indicate disease susceptibility, ancestry, and personal characteristics.” Items in the direct-to-consumer ancestry testing section were: “It would be interesting to know the risk of a certain disease”, “It would be interesting to know the risk of passing on a predisposition to a disease onto your children”, “It would be interesting to know my genetic ancestry”, “I would be concerned about the privacy of my genetic data”, “I would be concerned about the accuracy of the tests”, and “I would be concerned about how the test may impact my tribal enrollment”. The participants did not seem to relate direct-to-consumer testing with the items in the section, indicating the need to revise the statement to read: “The following questions refer to genetic testing companies. You may have seen commercials for these companies on TV. These companies provide a paid service that can give you information about certain health markers. Health markers could show disease risk, ancestry, and personal characteristics”. The items in the section were well understood except for the item using the phrase “predisposition to a disease”. All but one participant asked what the term predisposition meant during the cognitive interview and responded “strongly agree” to the item. The item was revised to read, “It would be interesting to know the risk of passing on a disease onto your children”. All other items were included without revision.

### 3.4. Culture and Spirituality

The next section of the survey was on culture and spirituality. The section began with the statement “The following questions address concerns that may or may not be relevant to your personal beliefs. Our team respects and embraces all persons’ beliefs. There are no right or wrong answers to these questions”. The section included a dozen statements about their views on culture and spirituality. The participants were provided with a brief introduction prior to the items stating, “Statements in this section were: “Religion and spirituality are important to understanding human health”; “Spirituality is important in my life”; “Body fluids and tissues are considered sacred or sensitive materials according to my beliefs”; “I would be willing to deposit body fluids and other tissues in a biobank”; “Anonymizing genetic material in a biobank may not protect the donor from spiritual harm”; “Biospecimens should have direct oversight by Elders or traditional healers while being used by a lab”; “Unused biospecimens should be returned to the Tribe”; “Labs should follow a Tribally approved ceremony when destroying samples”; “Developing “cell lines” that outlive the donor will result in spiritual implications.”; “Genetic research contradicts my spiritual beliefs”; “Indigenous rights should be recognized to repatriate biospecimens in the same way they can repatriate “human remains””; and “Anonymized samples sent to other labs may limit later attempts to repatriate Indigenous genetic materials”. The statement introducing the survey section was reduced in length to read “Personal beliefs. Our team respects and embraces all persons’ beliefs”.

Items in this section were removed due to participants having questions on the meaning of research definitions of “biobank”, “cell lines” and “anonymizing” or concepts like “repatriation”; however, the intention of the item was to have participants consider aspects of spirituality or spiritual practice which was not discussed in the think-aloud responses. Additionally, the participants tended to provide a neutral response on these items when a term was unclear, such as this participant who said “I couldn’t understand the question that’s why I put 4. What does anonymizing mean?”. Another issue that came to light was how the term “tribe” had complexity that was unaddressed in the item but necessary for participant consideration in coming to a response, again frequently resulting in a neutral response. One participant explained their considerations when thinking about “tribe”, “[I] wasn’t sure is it going to be returned by the Tribe or are they just going to destroy it. The Tribe—are we talking Eagle or Raven [clan], Tlingit or specifically Alaska Native [village]. That was a tricky question for me that’s why I picked 4”. Three items in which participants most clearly considered culture and spirituality and had a strong understanding of the wording of the item were kept for the final survey.

### 3.5. Items of Importance When Deciding to Participate in Research

An introduction statement was provided for the next section stating, “For the following statements, please indicate if you agree the following statements are important when you are making a decision to participate in a research study”. Nine items were included in this section: “Being able to access information researchers collect about me”; “That researchers keep my information private”; “Being able to control what kinds of research are done with my information”; “Being able to stop participating in the study”; “Knowing that I am contributing to new scientific discoveries”; “Being compensated for my time”; “Learning information about my health”; “Researchers who violate the informed consent should have serious consequences”; and “Having a contract signed by researchers”. The participants noted that the readability of the section would be improved if the root statement “taking part in a research study is important to” was present in addition to the stem statements. The item on having a research contract in place and compensation for participation had little participant discussion on the concepts of research contracts and compensation; so, the items were removed as they were not working as intended. Seven items in this section were kept for the final survey.

### 3.6. Specific Harms from Research Findings

The next survey section focused on specific harms from research findings. This section began with the root statement “It would be concerning if:” and was followed by six stem items: “Findings from the study were misleading, biased, or incorrect”; “Findings from the study would be used to advance political agendas”; “Data collected by the study would be used to harm me”; “Findings from the study would be used for profit”; “The government had my samples and information”; and “Researchers had my samples and information”. At two of the sites, the responses did not match the cognitive processes described by the participant and some participants described having issues with the root and stem of the question being separated. For example, when responding to the item on data being used to harm, a participant said, “I should have answered 7 after having it read with [to me with the] “it would be concerning” statement added.” None of the items from this section were included in the final survey.

### 3.7. Reconsent

A section on reconsent was included with a root question statement: “If you had to give consent for researchers to use your samples and information for each research project.” The four stem items related to reconsent were as follows: “I would feel bothered.”; “I would feel I would have control.”; “I would feel it was a waste of time.”; “I would not care how often I was asked for my permission.”. As with early sections that used separated root and stem items, the participant responses did not mirror their discussion on how they came to their response. None of the items from this section were included in the final survey.

### 3.8. Research Oversite Committee

A section on Tribal research oversight began with the premise statement for considering the items in this section: “If you had to give permission only one time at the beginning of the study for all research projects approved by an oversight committee”. The four items in this section were: “I would trust that oversight committee was making the right decisions about how to use my information”; “I would feel that researchers are making the best use of my information”; “If my personal information were removed, I would be willing to have my information and research results available on the internet to anyone”; and “Research participants and researchers should be equal partners in the study”. The item on research participants and researchers as equal partners was removed for several reasons: some participants felt they could not respond because the “study” was not specific, some participants felt the item was redundant, and other participants had suppositions on what equal partnership entailed that were not about power and control in research. Three items were kept for the final survey without edits.

### 3.9. Research Participant Involvement in the Research Process

Eight items were posed on research participant involvement in the research process: “Research participants should help design the study”, “Research participants should help choose what research questions to answer”, “Research participants should help decide what kinds of research are appropriate”, “Research participants should help recruit other participants”, “Research participants should help collect study data”, “Research participants should help analyze the data”, “Research participants should help decide what to do with study results”, and “If research participants helped plan and run the study, would that change your willingness to participate?”. The participants understood all items in this section, had responses that followed their think-aloud processes, and did not find these questions offensive; however, for several items, the participants had questions on the logistics surrounding the inclusion of research participants in the research team (e.g., the process used to select the research participants for research team inclusion, the tasks and training of research participants as research team members). When reflecting on the item on research participants helping in recruitment, a participant stated, “Simple to understand. I can participate in research by being a participant, but I don’t know or understand the underlying theory, don’t understand the meaning of the data or how to do the specific analysis so I might be able to help collect data but wouldn’t know what I was looking at”. The items on recruitment and helping to decide what to do with study results were kept for the final survey without edits.

### 3.10. Facets of Research That Are Important for Participants to Know

Five items were included in a section on important facets of a research project. This section was prefaced by the root statement “It is important to know …”. Stem items regarding research studies were as follows: “Who is using your health information for research”, “The types of research your health information is used for”, “If a security breach occurred”, “Who runs the study”, and “What would happen if a researcher misused the information”. The participants understood the wording of the statements and their responses mirrored their think-aloud statements. The five items were kept without changes for the final survey.

### 3.11. Concerns on Sharing Information

Six items were included in a section on concerns. The section was prefaced by the root statement “I am concerned with …”. Stem items related to issues of concern were as follows: “My financial information”, “My financial information”, “My health information”, “My genetic information”, “Information collected on my smart phone”, and “Information collected on a mobile device like a Fitbit”. The participants did not understand how the items were related to genetic research or other health research. None of the items from this section were included in the final survey.

### 3.12. Privacy

The statement “Do you agree or disagree with the following statements about privacy?” was presented before the five statements about privacy: “It is not possible to maintain my privacy these days”, “I limit my internet use because of privacy concerns”, “My largest privacy concern is identity theft”, “I don’t think there are enough legal protections of my privacy”, and “I limit the use of my smartphone because of privacy concerns.” The items in this section were found to be irritatingly broad, causing many participants to respond with a neutral response. When considering the item on legal protections, a participant said, “[there is] not enough information to answer. Broad question and depends on what ‘legal protections’ is”. None of the items from this section were included in the final survey.

### 3.13. Traits

A survey section on traits included the statement “To different degrees and in different ways, genetic factors and environmental factors contribute to an individual’s physical traits and psychological makeup. Do you agree that genes affect the characteristics listed below”. A matrix grid divided into sections on physical traits, psychological traits, cognitive traits, chronic disease, mental/behavioral health issues and beliefs was provided. Physical traits asked about were as follows: “height”, “eye color”, “Athletic ability”, and “most physical traits”. Psychological/cognitive traits asked about were as follows: “General Intelligence”, “Creative Talent”; “Temperament”; and “Most psychological/cognitive traits”. Chronic disease asked about was as follows: “Risk for Cardiovascular Disease”, “Risk for Diabetes”, “Most types of chronic diseases”. Separately, cancer was asked about with the following categories: “Risk for Breast Cancer”, “Risk for Lung Cancer”, and “Most types of cancer”. Mental/Behavioral health issues were: “Risk for Depression”, Risk for Suicide”, “Risk for Substance Abuse”, “Risk for Alcoholism”, and “Most mental/behavioral health issues”. Three beliefs were asked about, as follows: “Political Beliefs”, “Religious Beliefs” and “Most beliefs”. The participants reported multiple issues with the trait items. For example, some participants felt items within sections (e.g., “physical traits”) could have been asked as one item rather than individually, some disagreed with and/or felt the section on “mental/behavioral health issues” was offensive, and some participants were confused by the instructions on completing the matrix responses. Additionally, some participant survey responses were incongruent in relation to their think-aloud comments. Some participants noted that there were no items on the impacts of colonialism. None of the items from this section were kept for the final survey.

### 3.14. Potential Benefits of Genetic Testing

Four statements were presented in this section on potential benefits of genetic testing: “I would take a genetic test in order to learn about my ancestry/genealogy”; “I would take a genetic test in order to learn about my risk for preventable/treatable diseases”; “I would take a genetic test in order to learn about the risk my family members have for preventable/treatable diseases”; and “I would take a genetic test in order to gain information that helps me make decisions about whether and when to have children”. The item on using genetic test results for family planning was considered offensive as one participant called it a “disgusting question”. The remaining items were clearly understood with responses that mirrored the think-aloud processes and were not offensive. Three items on possible benefits of genetic testing were kept with no changes in the final survey.

### 3.15. Potential Risks of Genetic Testing

A section on the potential risks of genetic testing was prefaced with the statement “Genetic testing has several potential risks. To rate your level of concern for the potential risks listed below, circle the number that corresponds to your level of concern”. The four items in this section were as follows: “I would not take a genetic test because of the risk for a loss of privacy of my personal health information”; “I would not take a genetic test because of the risk for negative impacts on my health insurance coverage”; “I would not take a genetic test because of the risk for social stigmatization and/or the loss of my job”; “I would not take a genetic test because of uncertainty or anxiety about the test results”. All four items in the potential risks of genetic testing were kept for the final survey as the items were clearly understood with responses that mirrored the think-aloud processes and were not offensive.

### 3.16. Balancing Risks and Benefits of Genetic Testing

A section on balancing the potential benefits and potential harms of genetic testing included three items: “The potential benefits of taking a genetic tests outweigh the potential harms of taking a genetic test”; “After considering all the potential benefits and potential harms of genetic testing, I want to take a genetic test”; and “After considering all the potential benefits and potential harms of genetic testing, I want genetic testing to be available in my community”. Many participants disliked the repetitive language of each item and reported that the repetition of words made the item confusing. None of the items from this section were used in the final survey.

### 3.17. Genetic Testing at the Tribal Primary Care Clinic

A four-item section on genetic testing at the Tribal primary care clinic was included, as follows: “[site clinic name] should make genetic testing, pharmacogenetics, and other types of genetic medicine available to [site clinic term for patients]”; “[site clinic name] should conduct research on genetic testing, pharmacogenetics, and other types of genetic medicine”; “Making genetic testing, pharmacogenetics, and other types of genetic medicine available to [site clinic term for patients] should be a top priority of [site clinic name]”; and “[site clinic name] should expand clinical services and research related to genetic testing, pharmacogenetics, and other types of genetic medicine—even if that means reducing resources for other types of clinical services and other types of research”. Some participants mentioned not knowing the term “pharmacogenetics”, causing difficulty in selecting a response. A participant explained their response, “What is pharmacogenetics? I don’t know much about that, I’ll go neutral”. None of the items from this section were included in the final survey.

### 3.18. Community Benefits and Harms of Research

The five items in this section on the benefits and harms of research were as follows: “Participation in research can benefit individuals and communities”; “Participation in research can harm individuals and communities”; “The potential benefits of research outweigh the potential harms of research”; “After considering all the potential benefits and potential risks of research, I want to participate in research”; and “It is good for my community to participate in research”. Some participants felt the items on potential benefits outweighing potential harms and the item wanting to participate in research after considering all potential benefits and potential risks were overly vague and broad. One participant said, “Not telling me what type of research is going to be done. I’d need to know that first. Too generalized a question for me to answer one way or another” and responded with a neutral response. The remaining three items were well understood as written and the participants were able to respond to the items with the information provided in the item; the three items were included in the final survey.

### 3.19. Research Regulations

A five-item section on the perceptions of researchers and research regulations included the statements: “Research participants can trust researchers with their personal health information”; “Research participants can trust researchers to use their personal health information only as described in the informed consent documents”; “Research regulations adequately protect the privacy and autonomy of research participants”; “Researchers want to improve the health of research participants”, and “Researchers want to improve the health of communities participating in research”. The item concerning research regulations protecting privacy and autonomy was considered vague in regard to the content of ‘research regulations’, as one participant noted in their comment, “I don’t know what regulations govern research projects so I don’t know if they do adequately cover research participants” however the participant responded “agree”. Since there was a difference in their think-aloud comments and the item response, this question was not included in the final survey. The remaining four items were well understood as written and the participants were able to respond to the items with the information provided in the item; so, they were included in the final survey.

### 3.20. Individual Data Ownership

A seven-item section on data ownership included the following items: “I am willing to share my personal health data (including genomic data) for research purposes”; “I should be the owner of all of my personal health data (including genomic data)”; “As a member of an American Indian/Alaska Native tribe, I should share ownership of my personal health data (including genomic data) with my tribe or community”; “I want to be the owner of all of my personal health data (including genomic data)”; “I support research that may improve individual and community health, even if it puts individual and community privacy at risk”; “The right to own my genetic data is more important than the potential benefits of research”; and “AN/AI communities should have ownership of their community health information”. The survey respondents had a multitude of questions and expectations of individual data ownership. Given the variability in their think-aloud descriptions of data ownership at an individual level, we decided not to include any of the items from this section in the final survey.

### 3.21. Demographics

The final section of the survey was focused on participant characteristics. The section included fourteen items (with associated category response options): “How would you rate your overall health?” (Excellent, Very Good, Good, Fair, Poor); “What is your biological sex?” (Male; Female); “Do you have children?” (Yes, No); “Do you have grandchildren?” (Yes, No); “How old are you? (response: _ _ years); “How much school have you had?” (High school, GED, Some Vocational or Technical School, Technical School degree or certificate, Associate Degree, Some college, but no degree, Bachelor’s degree (BA, AB, BS), Graduate or Professional School (Masters, Doctorate, JD, DDS), Never attended school, Other: specify); “How many years have you received healthcare from [primary care location]?” (years); “The area I live in is:” (Rural, Suburban, Urban); “Have you ever participated in a research study?” (Yes, No); “Have you shared biological samples in a research study?” (Yes, No); “Have you shared biological samples in a biobank as part of participation in a research study?” (Yes, No); “I have sought information about genetics.” (Yes, No); “What race do you consider yourself to be?” (American Indian and/or Alaska Native, White, Black or African American, Asian, Native Hawaiian or Other Pacific Islander, Other:_[open ended text response]); “Are you Spanish, Hispanic, or Latino” (Yes, No). The item on the length of receipt of healthcare from the Tribal health location was removed as the respondents often considered the date of the Tribal health facility building for the location rather than the organization managing healthcare, which was the intention of the item. The item on rural, suburban or urban residence was removed as some respondents were unable to characterize their residence with the categories provided. Some participants had questions on what was included in the term “biological samples” in two of the items; so, in the item mentioning biological samples, a parenthetical addition was made, stating “biological samples (blood, urine, saliva)”. Otherwise, no additional issues were noted by participants and twelve items were kept for the final survey, including the three items on gender, race and ethnicity that are commonly used for National Institutes of Health study enrollment reporting.

### 3.22. Comments on Cognitive Interviewing

Following the retrospective cognitive interview, the participants were asked if they had any final comments. Surprisingly, the participants commented that they appreciated being asked to help shape a survey that would be used with AIAN people as the cognitive interviewing process showed the intention on validating survey items for AIAN people as the participants endorsed the methodology with comments like “[it is ] good that research is looking into how people answer surveys, what the wording is on these surveys”, “It’s good to know what we think!” and “I sure appreciate you guys doing this”. Another participant described their thoughts on testing items of cultural relevance as they said “I think it’s ok. I hope it helps somebody. It was hard. I like how you guys asked for our permission and about the spiritual”.

### 3.23. Final Instrument

Several scales were not included in the final CEIGR cross-site survey. Within scales, survey items were revised or removed based on participant responses. Over half of the items (58%) were determined to need at least one revision based on cognitive testing findings. Following discussion with the three CEIGR sites, 52 items were finalized for a survey on genetics in AIAN communities to be used in future efforts.

## 4. Discussion

Our team primarily used a think-aloud methodology to gather AIAN individuals’ input on genetics related survey items in three locations in the US with populations representing a multitude of Tribal nations. The CI process we used was effective in identifying instruction, survey item and response option issues in newly developed and existing instruments. By testing the draft survey in multiple locations in the US with people from many AIAN tribes, we were able to identify variation in the functioning of an item or scale between groups. Had we not conducted cognitive testing and removed or revised items, we would have introduced systematic bias that could impact meaningful empiric information on genetic research practice and preferences among AIAN people.

Our findings contribute to a sparse literature on cognitive testing among AIAN populations, as only four papers describing health survey cognitive testing are available despite surveys being commonly used to describe AIAN health status [43,45,46,47]. Like those of Dillard et al., 2023, Gunville and Williams, 2019, Pavkov et al., 2012, and Weidmer-Ocampo et al., 2009, our findings reinforce the importance of performing CIs prior to survey deployment. For example, over half of the items we tested needed at least one type of revision. Further, we found cross-site variability in the comprehension and in the use of a neutral response when there was uncertainty in comprehension or interest in a better description of the context for the application of the item.

Future research is needed on the use of a neutral response in AIAN populations as a manifestation of aspects of non-interference. Non-interference in north American Indigenous peoples is a complex and flexible concept based on numerous contextual factors, not a prohibition of any form of coercion or interference [48]. In our sample, some participants expressed facets of non-interference when considering items as they described aspects of proscriptive and prescriptive norms of living as a human being, respect for individual self-determination, Tribal self-determination and minimizing strife among family members.

The CI process is a method to validate a survey instrument’s face validity and enhance confidence in item interpretation, as well as a method to engage communities in the research process [43]. Although not a primary outcome of our study, the participants had positive feedback on the CI process as a form of engagement with AIAN communities where their input directly influences the research being conducted in their communities. The CI process also provided community members with an awareness of the topics being considered and could be used as a method that contributes to the notion of community protection with community members’ in-depth feedback being analyzed and considered.

### Strengths and Limitations

This study had some limitations as we did not recruit a stratified sample of the overall population. In consideration of the large number of AIAN tribes and geographic distribution of AIAN people, this study was limited to a small proportion of AIAN communities. This study was carried out with a single round of cognitive interviewing at all sites. However, the large number of participants (*n* = 52) and multiple responses per item across the sample allowed for a rich description of cognitive and cultural issues per item. For context, the initial surveys for the precision medicine national cohort study of a million people, the All of Us Research Project, consisted of only 74 participants in the first round of cognitive testing [49]. Additionally, our sample included both urban, and rural reservation-based AIAN adults and both AI and AN people. Although the sample had equal numbers of men and women, 54% reported living in a rural area, and the population was skewed towards younger ages, much like the overall AIAN population. Our sample reported having a higher education level and thus the findings may not generalize to the AIAN population who do not have a college education.

## 5. Conclusions

The composite cross-site survey was revised to include items with high AIAN cross-site comprehensibility and applicability to AIAN primary care settings. The items included in the final survey are intended as a tool for empirical research that Tribal leaders and their partners can use to develop, enhance and reinforce genetic policy, practices and approaches that are community-appropriate. It was the intention of the CEIGR to put forward an instrument that could be used within AIAN healthcare and public health settings to guide policymakers and healthcare providers in developing genetics, genomics and precision medicine approaches for AIAN community members.

## Figures and Tables

**Table 1:** Examples of problems identified through cognitive interviews and resolution.

Initial Item and Response	Example Participant Response	Reason for Modification	Updated Item
Item: It would be interesting to know the risk of passing a predisposition to a disease onto your children. *	“Predisposition, what does that mean?”	Multiple participants not understanding key term within item	I would pay for genetic testing to know the risk of passing a disease onto my children.
Item: Have you shared biological samples in a research study? *	“Biological you mean like human blood, tissue body fluids?”	Multiple participants uncertain about what “biological sample” term is includes	“Have you shared biological (blood, urine, saliva) samples in a research study?”
Item: Anonymizing genetic material in a biobank may not protect the donor from spiritual harm. *	“Did I put 4? What does this mean? I couldn’t understand the question that’s why I put 4. What does anonymizing mean to you?”	Multiple neutral item responses when participants did not understand a term in the item. Multiple issues with terms “anonymizing”, “genetic material”, “donor”, “spiritual harm”.	Remove item
Item: The area I live in is Responses: Rural, suburban, urban	“Am I urban or suburban?”	Confusion as to delineating response categories provided	Remove item

* Response option was a seven-point Likert scale with response anchors of 1 = strongly disagree, 7 = strongly agree.

## Data Availability

The data presented in this study are available on request from the corresponding author due to Tribal research codes of participating Tribal organizations.
